# Evaluation of the association of length of stay in hospital and outcomes

**DOI:** 10.1093/intqhc/mzab160

**Published:** 2021-12-17

**Authors:** Thang S Han, Paul Murray, Jonathan Robin, Peter Wilkinson, David Fluck, Christopher H Fry

**Affiliations:** Department of Endocrinology, Ashford and St Peter’s Hospitals NHS Foundation Trust, Guildford Road, Chertsey, Surrey KT16 0PZ, UK; Institute of Cardiovascular Research, Royal Holloway, University of London, Egham Hill, Egham, Surrey TW20 0EX, UK; Department of Respiratory Medicine, Ashford and St Peter’s Hospitals NHS Foundation Trust, Guildford Road, Chertsey, Surrey KT16 0PZ, UK; Acute Medical Unit, Ashford and St Peter’s Hospitals NHS Foundation Trust, Guildford Road, Chertsey, Surrey KT16 0PZ, UK; Department of Cardiology, Ashford and St Peter’s Hospitals NHS Foundation Trust, Guildford Road, Chertsey, Surrey KT16 0PZ, UK; Department of Cardiology, Ashford and St Peter’s Hospitals NHS Foundation Trust, Guildford Road, Chertsey, Surrey KT16 0PZ, UK; School of Physiology, Pharmacology and Neuroscience, University of Bristol, Biomedical Sciences Building, University Walk, Bristol BS8 1TD, UK

**Keywords:** getting it right first time, quality management, measurement of quality, health economy, mortality

## Abstract

**Background:**

There exist wide variations in healthcare quality within the National Health Service (NHS). A shorter hospital length of stay (LOS) has been implicated as premature discharge, that may in turn lead to adverse consequences. We tested the hypothesis that a short LOS might be associated with increased risk of readmissions within 28 days of hospital discharge and also post-discharge mortality.

**Methods:**

We conducted a single-centred study of 32 270 (46.1% men) consecutive alive-discharge episodes (mean age = 64.0 years, standard deviation = 20.5, range = 18–107 years), collected between 01/04/2017 and 31/03/2019. Associations of LOS tertiles (middle tertile as a reference) with readmissions and mortality were assessed using observed/expected ratios, and logistic and Cox regressions to estimate odds (OR) and hazard ratios (HR) (adjusted for age, sex, patients’ severity of underlying health status and index admissions), with 95% confidence intervals (CIs).

**Results:**

The observed numbers of readmissions within 28 days of hospital discharge or post-discharge mortality were lower than expected (observed: expected ratio < 1) in patients in the bottom tertile (<1.2 days) and middle tertile (1.2–4.3 days) of LOS, whilst higher than expected (observed: expected ratio > 1) in patients in the top tertile (>4.3 days), amongst all ages. Patients in the top tertile of LOS had increased risks for one readmission: OR = 2.32 (95% CI = 1.86–2.88) or ≥2 readmissions: OR = 6.17 (95% CI = 5.11–7.45), death within 30 days: OR = 2.87 (95% CI = 2.34–3.51), and within six months of discharge: OR = 2.52 (95% CI = 2.23–2.85), and death over a two-year period: HR = 2.25 (95% CI = 2.05–2.47). The LOS explained 7.4% and 15.9% of the total variance (*r*^2^) in one readmission and ≥2 readmissions, and 9.1% and 10.0% of the total variance in mortality with 30 days and within six months of hospital discharge, respectively. Within the bottom, middle and top tertiles of the initial LOS, the median duration from hospital discharge to death progressively shortened from 136, 126 to 80 days, whilst LOS during readmission lengthened from 0.4, 0.9 to 2.8 days, respectively.

**Conclusion:**

Short LOS in hospital was associated with favourable post-discharge outcomes such as early readmission and mortality, and with a delay in time interval from discharge to death and shorter LOS in hospital during readmission. These findings indicate that timely discharge from our hospital meets the aims of the NHS-generated national improvement programme, Getting It Right First Time.

## Introduction

The length of stay (LOS) in hospital has been a focus of research in quality of clinical care and efficiencies, mostly in older adults living with chronic conditions [1]. Information on the LOS and associated outcome measures are important, more now than ever since the number of acute admissions and readmissions have been rising steadily over recent years [2], in parallel to the expanding ageing population [3]. Within the National Health Service (NHS), there exist wide variations in healthcare quality measures including LOS, readmission rates and mortality [2]. Getting It Right First Time (GIRFT) was thus created recently by the NHS to address this issue. This national improvement programme, led by frontline clinicians, helps identify and reduce unwarranted variations in service and practice, aiming to improve the quality of medical and clinical care, patient outcomes and efficiencies [4, 5].

Instead of waiting for medical issues to be resolved before planning social and care provision in the community, discharge planning from an early stage of admission, involving multidisciplinary team efforts, has been recommended as the key to timely discharge, which is important both to the patient and the healthcare service [6]. How early an appropriate discharge should be has been a major topic of debate and closely scrutinised. Studies have found inconsistent evidence on the association of the LOS and outcomes, for example, early readmissions in patients with chronic conditions such as congestive heart failure [6], and post-discharge mortality in patients who sustained a hip fracture [7].

Because of such conflicting findings, short LOS has been implicated as premature discharge that could lead to adverse health consequences. We therefore tested the hypothesis that shorter LOS in hospital might be associated with the risk of adverse outcomes, including all-cause frequent early readmissions and all-cause mortality after a discharge in adults 18–107 years, taking into account age, sex, underlying health status and index admissions.

## Methods

### Design, participants and setting

Data were collected on consecutive alive–discharge episodes over two years between 1 April 2017 and 31 March 2019 in an NHS District General Hospital located in the outskirts of London serving a population of 400 000 [8, 9]. Information was recorded using the Patient Administration System implemented by the NHS including: demographics, diagnosis, co-morbidities, dates of emergency (non-elective) admission and readmission and date of death after discharge. From these data were calculated: the frequency of readmissions within 28 days of hospital discharge; as well as mortality within 30 days and six months after hospital discharge, and over a two-year period. This study included all adults from the age of 18 admitted over the two-year period of study without any age restriction (oldest aged 107 years). Cancer and obstetrics admissions were not included, in line with the NHS data collection protocol for emergency hospital admissions [10].

### Indicators of severity of underlying health status

Underlying health status was indicated by index admissions, acuity of admission (emergency or elective admission), comorbidity of the patient and use of the emergency department in the six months before admission. Index admissions and morbidities were coded according to the international classification of diseases (ICD-11) [11] for calculation of the Charlson co-morbidity index (CCI) [12].

### Categorization of variables

Since there exist no defined cut-offs for LOS, this was therefore grouped into tertiles: bottom (<1.2 days), middle (1.2–4.3 days) and top tertiles (>4.3 days). The frequency of readmissions within 28 days of hospital discharge was categorized into three groups: no readmission, one readmission and ≥2 readmissions. Five age bands were created: 18–49, 50–59, 60–69, 70–79 and ≥80 years. Individuals aged 18–49 years were combined together due to low mortality rates, and patients aged 80–107 years were grouped together due to small numbers of patients. Acuity of admission and emergency department use were categorized into variables according to the absence or presence of the factor. There were 29 958 (92.8%) patients who had a CCI score = 0, and of the remainder 2005 (6.2%), 305 (0.9%) and 2 patients had respective CCI scores of 1, 2 and 6. We therefore categorized CCI into two groups for analysis: CCI score = 0 and CCI score ≥1.

### Statistical analysis

Chi-squared tests were used to explore the associ-ation between categorical variables. Receiver operating characteristic (ROC) curves were constructed to determine the area under the curve (AUC) for the LOS as a predictor of outcomes (frequent readmissions or mortality). The middle tertile of LOS was used as the reference group to: assess the association of shorter (bottom tertile) and longer (top tertile) periods with the time from discharge to death using Kruskal–Wallis H tests; predict readmission frequency and mortality within 30 days and within six months of hospital discharge using observed/expected ratios and multivariable logistic regression; and predict mortality over two years (a time-dependent event) using Cox regression. Data were presented in three models: (i) unadjusted; (ii) adjusted for age and sex and (iii) adjusted for age, sex, index admissions (see Supplementary Table S1 and S2 for complete list) and severity of underlying health status of the patient (acute admission, CCI and emergency department use). Data are reported as odds ratios (ORs) and hazard ratios (HRs), with 95% confidence intervals (CIs) and variances *r*^2^) for the entire sample and for individual age groups. Analyses were performed using IBM SPSS Statistics, v25.0 (IBM Corp., Armonk, NY).

## Results

### Subject characteristics

Data from a total of 14 878 men and 17 392 women of mean age 64.0 and standard deviation = 20.5 years were analysed. There were 72.3% of elective and 27.7% of non-elective admissions. There were 88.5% of patients who were not readmitted, 8.3% readmitted once and 3.3% readmitted ≥2 times within 28 days of discharge. The mortality rates within 30 days and six months of discharge from the first admission were 2.6 and 6.8% and increased to 10.2% over the two-year period. The median and interquartile range (IQR) LOS was 0.5 days (0.2–1.0), 2.4 days (1.8–3.2) and 9.3 (6.3–16.9) days for patients in the bottom, middle and top tertiles of LOS, respectively. Patient characteristics including age distribution and frequency of index admissions are shown in Supplementary Table S1.

### Receiver operating characteristic analysis

ROC analysis to generate AUC values showed that the LOS as a predictor of a single readmission was 69.0% (95% CI = 60.0–70.1, *P* < 0.001), ≥2 readmissions was 82.5% (95% CI = 81.5–63.6, *P* < 0.001) and all mortality was 72.2% (95% CI = 71.3–73.2, *P* < 0.001) (Supplementary Figure S1).

### Index admissions in relation to initial LOS

The highest proportions of index admissions in the top tertile of a LOS were found amongst patients admitted with congestive heart failure, neurological disorders, chronic obstructive pulmonary disease, pneumonia, psychiatric disorders, dermatological disorders, sepsis and bone fractures. Conversely, the proportions of index admissions amongst top tertiles lower than those in the middle tertile of LOS included myocardial infarct, atrial fibrillation, asthma, gastrointestinal disorders, endocrine disorders, rheumatological disorders, haematological disorders, genitourinary disorders, viral infections, ophthalmic disorders, non-specific pain and medical device-related complications (Supplementary Table S2).

### Rates of readmissions and mortality based on initial LOS

**Figure 1:**
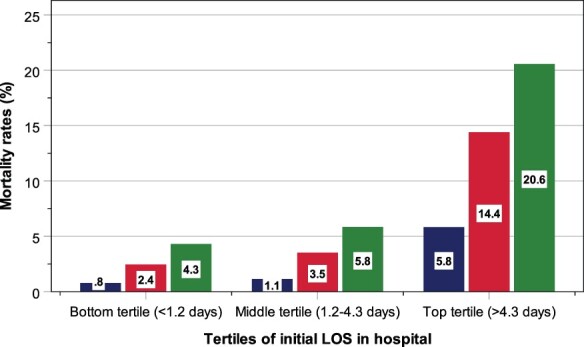
Rates of mortality within 30 days (blue bars) and six months of discharge (red bars), as well as over a two-year period (green bars), according to tertiles of initial LOS in hospital.

Among patients in the bottom, middle and top tertiles, the rates of one readmission were 3.5, 6.0 and 15.3% and ≥2 readmissions were 0.2, 1.3 and 8.3%, respectively. The corresponding rates of mortality within 30 days of discharge were 0.8, 1.1 and 5.8%, and within six months of discharge were 2.4, 3.5 and 14.4%, and increased further to 4.3, 5.8 and 20.6% over the two-year period (Figure 1). These increases were observed in all patients ranging from the youngest (aged 19–49 years) to the oldest (aged ≥80 years) group. The proportions of acute admission, CCI scores ≥ 1, and emergency department visits within six months, as well as a number of major index diagnoses were increased with longer hospital LOS (Table 1).

**Table 1:** Age-specific rates of readmissions with 28 days and mortality within 30 days or six months and over two years of hospital discharge, according to the initial length of stay in hospital in 32 270 alive discharge episodes

	Rates (%)			Group differences	
	Bottom tertile (*n* = 10 754)	Middle tertile (*n* = 10 757)	Top tertile (*n* = 10 759)	χ^2^	*P*
Readmissions within 28 days					
18–49 years: 1 readmission	3.3	5.1	11.3	259.4	<0.001
18–49 years: ≥2 readmissions	0.2	1.1	4.3		
50–59 years: 1 readmission	2.8	5.1	12.5	206.2	<0.001
50–59 years: ≥2 readmissions	0.2	0.9	4.9		
60–69 years: 1 readmission	3.1	5.7	11.9	249.9	<0.001
60–69 years: ≥2 readmissions	0.1	1.1	6.4		
70–79 years: 1 readmission	3.2	4.7	14.1	425.1	<0.001
70–79 years: ≥2 readmissions	0.2	1.2	7.4		
≥80 years: 1 readmission	5.5	9.4	18.2	574.4	<0.001
≥80 years: ≥2 readmissions	0.5	2.2	10.7		
All ages: 1 readmission	3.5	6.0	15.3	2538.5	<0.001
All ages: ≥2 readmissions	0.2	1.3	8.3		
Died within 30 days					
18–49 years	0.0	0.1	1.1	51.5	<0.001
50–59 years	0.3	0.4	1.9	27.8	<0.001
60–69 years	0.8	1.1	3.6	40.1	<0.001
70–79 years	1.4	1.6	5.1	69.2	<0.001
≥80 years	2.6	3.0	8.5	124.5	<0.001
All ages	0.8	1.1	5.8	6771	<0.001
Died within six months					
18–49 years	0.2	0.3	2.5	89.1	<0.001
50–59 years	0.8	1.1	5.8	84.7	<0.001
60–69 years	2.1	2.6	10.5	142.7	<0.001
70–79 years	3.8	4.3	13.2	168.1	<0.001
≥80 years	8.7	10.3	20.2	187.3	<0.001
All ages	2.4	3.5	14.4	1487.7	<0.001
Died over two years					
18–49 years	0.5	0.5	3.6	99.6	<0.001
50–59 years	1.5	1.9	8.5	111.4	<0.001
60–69 years	3.1	4.4	13.9	163.4	<0.001
70–79 years	7.1	7.4	20.1	222.5	<0.001
≥80 years	15.2	16.8	28.5	187.2	<0.001
All ages	4.3	5.8	20.6	1886.4	<0.001
Underlying health status					
Acute admission	64.3	67.1	85.5	1422	<0.001
CCI score = 0	96.7	93.5	88.3	606.1	<0.001
CCI score = 1	2.7	5.4	10.5		
CCI score = 2	0.7	1.0	1.2		
ED visits within six months	77.3	72.6	88.8	923	<0.001
Selected index admissions					
Congestive heart failure	0.4	0.9	2.5	395.4	<0.001
Pneumonia	1.9	4.7	9.1	568.3	<0.001
Stroke	0.7	1.9	5.4	502.6	<0.001
Diabetes	0.8	1.1	1.3	13.2	<0.001
Sepsis	1.7	3.3	5.9	282.8	<0.001
Urinary tract infection	2.2	3.0	5.1	147.2	<0.001

CCI, Charlson comorbidity index; ED, emergency department.

### Risk of early readmissions based on initial LOS

The observed numbers of readmissions within 28 days of hospital discharge were lower than expected (observed/expected ratio <1) in patients in the bottom and middle tertiles of LOS throughout all age categories (except a slightly higher ratio for those below 50 years in middle tertile). On the contrary, the observed numbers of readmissions were higher than expected (observed/expected ratio >1) in patients in the top tertile of LOS amongst all ages (Table 2). Logistic regression analysis showed that compared with patients with a LOS in the middle tertile, those in the top tertile of LOS were associated with increased risk of one readmission: adjusted OR = 2.30 (95% CI = 1.87–2.83) or ≥2 readmissions: adjusted OR = 5.47 (95% CI = 4.53–6.61), whilst those in the bottom tertile of LOS were associated with a reduced risk for readmission. The LOS explained 7.4% and 15.9% of the total variance (*r*^2^) in readmission once and two or more times, respectively. These corresponding variances increased to 8% and 16.9% with the addition of age and sex and further to 12.5% and 21.5% with the addition of patients’ severity of underlying health status and index admissions (Table 3A).

**Table 2:** Age-specific rates of readmissions with 28 days and mortality within 30 days or six months, and over two years of hospital discharge, according to the initial length of stay in hospital in 32 270 alive discharge episodes

	Bottom tertile (*n* = 10 754)		Middle tertile (*n* = 10 757)		Top tertile (*n* = 10 759)	
	O/E (*n*)	O/E ratio	O/E (*n*)	O/E ratio	O/E (*n*)	O/E ratio
Readmissions within 28 days						
18–49 years: 1 readmission	142/212.6	0.67	156/151.8	1.03	118/51.5	2.29
18–49 years: ≥2 readmissions	9/44.5	0.20	33/31.8	1.04	45/10.8	4.17
50–59 years: 1 readmission	47/96.7	0.49	87/99.5	0.87	117/54.8	2.14
50–59 years: ≥2 readmissions	3/25.1	0.12	16/25.8	0.62	46/14.2	3.24
60–69 years: 1 readmission	46/100.4	0.46	104/122.5	0.85	167/94.0	1.78
60–69 years: ≥2 readmissions	2/35.5	0.06	20/43.3	0.46	90/33.2	2.71
70–79 years: 1 readmission	51/129.2	0.39	97/164.6	0.59	337/191.2	1.76
70–79 years: ≥2 readmissions	3/54.1	0.06	24/68.9	0.35	176/80.0	2.20
≥80 years: 1 readmission	93/229.9	0.40	197/286.1	0.69	907/680.9	1.33
≥80 years: ≥2 readmissions	9/113.1	0.08	45/140.8	0.32	535/335.1	1.60
Died within 30 days						
18–49 years	2/7.7	0.26	2/5.5	0.36	11/1.9	5.79
50–59 years	5/11.2	0.45	6/11.5	0.52	18/6.3	2.86
60–69 years	12/26.6	0.45	21/32.5	0.65	51/24.9	2.05
70–79 years	22/47.2	0.47	32/60.1	0.53	123/69.8	1.76
≥80 years	43/101.6	0.42	62/126.5	0.49	424/300.9	1.41
Died within six months						
18–49 years	9/22.5	0.40	9/16.1	0.56	26/5.5	4.73
50–59 years	14/33.5	0.42	19/34.5	0.55	54/19.0	2.84
60–69 years	32/71.6	0.45	47/87.4	0.54	147/67.1	2.19
70–79 years	62/124.2	0.50	89/158.1	0.56	315/183.7	1.71
≥80 years	146/263.0	0.56	215/327.2	0.66	1008/778.8	1.29
Died over two years						
18–49 years	22/37.3	0.59	14/26.6	0.53	37/9.0	4.11
50–59 years	25/52.8	0.47	32/54.3	0.59	80/29.9	2.68
60–69 years	46/102.0	0.45	80/124.5	0.64	196/95.5	2.05
70–79 years	114/199.1	0.57	152/253.5	0.60	481/294.5	1.63
≥80 years	256/389.2	0.66	351/484.3	0.72	1419/1152.5	1.23

O/E, observed/expected.

**Table 3:** Risk of readmissions within 28 days and mortality within 30 days and within six months of discharge from hospital (A) and over a two-year period (B) according to the initial legth of stay in hospital in 32 270 alive discharge episodes

	Risk of readmissions and mortality											
	Unadjusted				Adjusted for age and sex				Adjusted for age, sex, patients’ severity^b^ and index admissions^c^			
Table 3A	OR	95% CI	*r* ^2^ (%)	*P*	OR	95% CI	*r* ^2^ (%)	*P*	OR	95% CI	*r* ^2^ (%)	*P*
1 readmission												
Bottom tertile of LOS	0.32	0.21–0.49	7.4	<0.001	0.33	0.21–0.51	8.0	<0.001	0.32	0.21–0.50	12.5	<0.001
Middle tertile of LOS^a^	1	–		–	1	–		–	1	–		–
Top tertile of LOS	2.52	2.16–3.08		<0.001	2.30	1.97–2.83		<0.001	2.32	1.88–2.88		<0.001
≥2 readmissions												
Bottom tertile of LOS	0.18	0.12–0.28	15.9	<0.001	0.20	0.13–0.30	16.9	<0.001	0.18	0.12–0.28	21.5	<0.001
Middle tertile of LOS^a^	1	–		–	1	–		–	1	–		–
Top tertile of LOS	7.85	6.54–9.41		<0.001	6.21	5.16–7.49		<0.001	6.17	5.11–7.45		<0.001
Died within 30 days												
Bottom tertile of LOS	0.68	0.52–0.90	9.1	0.007	0.81	0.61–1.07	12.5	0.143	0.86	0.64–1.14	15.4	0.292
Middle tertile of LOS^a^	1	–		–	1	–		–	1	–		–
Top tertile of LOS	5.35	4.40–6.50		<0.001	3.28	2.69–4.01		<0.001	2.87	2.34–3.51		<0.001
Died within 6 months												
Bottom tertile of LOS	0.69	0.59–0.91	10.8	<0.001	0.82	0.70–0.96	14.2	0.016	0.83	0.71–0.99	17.3	0.032
Middle tertile of LOS^a^	1	–		–	1	–		–	1	–		–
Top tertile of LOS	4.61	4.11–5.18		<0.001	2.83	2.51–3.19		<0.001	2.52	2.23–2.85		<0.001
	Risk of mortality over two year period											
	Unadjusted				Adjusted for age and sex				Adjusted for age, sex, patients’ severity^b^ and index admissions^c^			
Table 3B	OR	95% CI		*P*	OR	95% CI		*P*	OR	95% CI		*P*
All ages: Bottom tertile of LOS	0.73	0.65–0.82		<0.001	0.86	0.77–0.97		0.017	0.86	0.76–0.97		0.016
All ages: Middle tertile of LOS^a^	1	–		–	1	–		–	1	–		–
All ages: Top tertile of LOS	3.89	3.56–4.26		<0.001	2.34	2.14–2.57		<0.001	2.25	2.05−2.47		<0.001
18–49 years: Bottom tertile of LOS	1.13	0.58–2.20		0.730	1.19	0.61–2.33		0.613	1.27	0.64–2.53		0.431
18–49 years: Middle tertile of LOS^a^	1	–		–	1	–		–	1	–		–
18–49 years: Top tertile of LOS	7.97	4.31–14.74		<0.001	7.21	3.89–13.36		<0.001	6.67	3.54–12.56		<0.001
50–59 years: Bottom tertile of LOS	0.80	4.48–1.35		0.405	0.82	0.48–1.38		0.445	0.72	0.42–1.23		0.231
50–59 years: Middle tertile of LOS^a^	1	–		–	1	–		–	1	–		–
50–59 years: Top tertile of LOS	4.78	3.17–7.20		<0.001	4.75	3.15–7.16		<0.001	4.18	2.74–6.37		<0.001
60–69 years: Bottom tertile of LOS	0.70	0.49–1.00		0.052	0.70	0.49–1.01		0.058	0.62	0.43–0.90		0.013
60–69 years: Middle tertile of LOS^a^	1	–		–	1	–		–	1	–		–
60–69 years: Top tertile of LOS	3.40	2.62–4.41		<0.001	3.37	2.59–4.37		<0.001	3.09	2.37–4.03		<0.001
70–79 years: Bottom tertile of LOS	0.95	0.74–1.21		0.648	0.94	0.74–1.20		0.624	0.88	0.69–1.13		0.309
70–79 years: Middle tertile of LOS^a^	1	–		–	1	–		–	1	–		–
70–79 years: Top tertile of LOS	2.98	2.48–3.58		<0.001	2.95	2.45–3.54		<0.001	2.72	2.26–3.27		<0.001
≥80 years: Bottom tertile of LOS	0.89	0.76–1.04		0.145	0.89	0.76–1.05		0.162	0.93	0.79–1.09		0.365
≥80 years: Middle tertile of LOS^a^	1	–		–	1	–		–	1	–		–
≥80 years: Top tertile of LOS	1.86	1.66–2.09		<0.001	1.81	1.61–2.03		<0.001	1.77	1.58–2.00		<0.001

OR, odds ratio; LOS, length of stay;.

a Reference group = middle tertile;

b Patients’ severity: Acuity of admission, Charlson Comorbidity Index score ≥1 and emergency department use;

c See Supplementary Table S2 for complete list of index admissions.

### Risk of mortality within 30 days and six months of discharge based on the LOS

The observed numbers of mortality within 30 days and within six months of hospital discharge were lower in patients with LOS in the bottom and middle tertiles and were higher than expected in those with LOS in the top tertile throughout all ages (Table 2). Compared with patients with a LOS in the middle tertile, patients who stayed longest in hospital (top tertile of LOS) were associated with increased risk of death within 30 days of discharge: adjusted OR = 2.87 (95% CI = 2.34–3.51), and within six months of discharge: OR = 2.52 (95% CI = 2.23–2.85). By contrast, those in the bottom tertile of LOS did not differ in the risk death within 30 days of discharge: age and sex adjusted OR = 0.86 (95% CI = 0.64–1.14), but did have a significantly reduced risk of death within six months of discharge from hospital: OR = 0.83 (95% CI = 0.71–0.99). The LOS explained 9.1% and 10.0% of the total variance (*r*^2^) in mortality with 30 days and within six months of hospital discharge, respectively. These corresponding variances increased to 12.5% and 14.2% with the addition of age and sex and further to 15.4% and 14.3% with the addition of patients’ severity and index admissions (Table 3A).

### Risk of mortality over a two-year period based on the LOS

**Figure 2:**
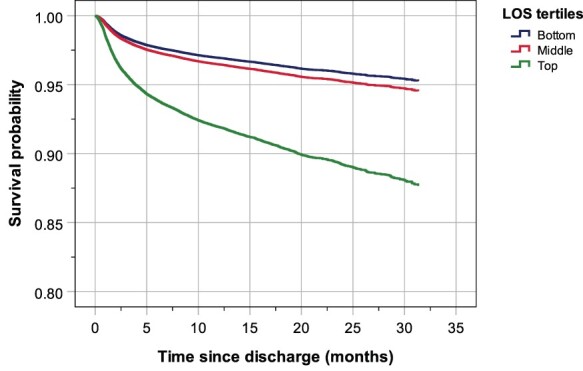
Survival probability, adjusted for age and sex, of patient of in different tertiles of LOS in hospital. The number of patients exposed to risk is shown in Supplementary Table S3.

After adjustment for age and sex, survival plot revealed that the survival probability was lower with increasing LOS (Figure 2). Multivariable Cox regression showed that over a two-year period, compared with patients in the middle tertile of a LOS, the risk of mortality was higher for those who stayed the longest time (top tertile of LOS) in hospital: adjusted HR = 2.25 (95% CI = 2.05–2.47), and was lower for patients who stayed the shortest time (bottom tertile of LOS): adjusted HR = 0.86 (95% CI = 0.76–0.97). Analysis according to age stratification showed that compared with middle tertile of the LOS, the adjusted HRs were higher amongst the younger age groups in the top tertile of LOS. There were no statistical differences in mortality between middle and bottom tertiles of LOS, except a significantly lower risk amongst the 60–69 year group: adjusted HR = 0.62 (95% CI = 0.43–0.90) (Table 3B).

### Time from discharge to death and LOS during readmission based on initial length of stay

**Figure 3:**
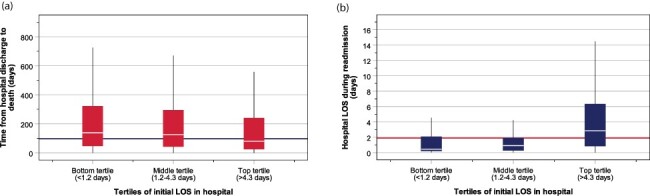
Box-and-whisker plot showing the median and interquartile range of the time from discharge to death (a) and LOS during readmission (b). Horizontal lines indicate grand median values.

Among 3305 patients who died after hospital discharge, the median time taken from discharge to death was 97 days (IQR = 30–259) and was significantly different between tertiles of LOS (Kruskal–Wallis H test for group differences: χ^2^ = 61.2, *P* < 0.001). The median (IQR) time from discharge to death was 126 days (43–294) for patients in the middle tertile of LOS, which was significantly (*P* < 0.001) delayed to 138 days (48–323) for those who spent the shortest time in hospital (bottom tertile of LOS) and was significantly (*P* < 0.001) curtailed to 80 days (26–239) for those who stayed longest in hospital (top tertile of LOS) (Figure 3a). During readmission, the group median LOS stay was 1.9 days (IQR = 0.5–5.4) and differed between the tertiles (Kruskal–Wallis H test: χ^2^ = 139.3, *P* < 0.001). The LOS was shortest amongst those with initial LOS in the bottom tertile: median = 0.4 days (0.2–2.2), followed middle tertile: 0.9 days (0.3–1.9) and longest 2.8 days (0.8–6.3) (Figure 3b).

## Discussion

### Statement of principal findings

Based on our analysis of 32 270 adults, aged between 18 and 107 years, two significant findings emerged. First, shorter LOS was not associated with an increase in the risk of frequent readmissions or mortality in patients after discharge from hospital. This indicates a timely discharge and a shorter LOS therefore does not equate to premature discharge. Second, a longer LOS is associated with increased risk of frequent readmissions and mortality. Our findings provide supportive evidence of good clinical practice that fulfils the concept of the GIRFT programme [4, 5] and valuable data for future quality of care assessment within this centre and other NHS hospitals.

### Interpretation within the context of the wider literature

The results observed in this study show that the test accuracies of the LOS to predict outcome measures were high for ≥2 readmissions within 28 days of discharge and moderate for all-cause mortality. Other factors may also contribute to this association include index admissions and patients’ severity of underlying illness, as reflected by higher proportions of acuity of admission, CCI score ≥1 and emergency department visits within six months among patients with LOS in the top tertile.

Existing research on the LOS in hospital and consequences after discharge from hospital has focussed primarily on older adult populations [6, 13]. Our findings of the association of a longer LOS in hospital with increased risk of readmissions and death are consistent with findings from previous studies [6, 7], while the observations that outcomes in patients with shorter LOS were better or no worse are reassuring. These associations were present in all age groups. As far as we are aware, this type of analysis, particularly in younger individuals, performed in our study is not readily available in the published literature.

Readmissions impose a heavy burden on healthcare services. In the USA, costs vary between $8500 and $9500 for readmissions with respiratory or cardiac events, rising to $10 000 for readmission with sepsis [14] and $13 500 for coronary artery bypass grafts [15], totalling $26 billion a year (in 2019?) [16]. In England, emergency admissions cost the NHS £11 billion ($15 billion), with 5% coming from those readmitted within 30 days of hospital discharge [17]. Studies have examined the level of social and healthcare support to reduce readmissions. A recent study has shown that early readmission rates could be lowered by a simple telephone contact with patients within 48 h of discharge [18], while another showed that a post-discharge care bundle provided to patients (which consisted of medication reconciliation by a clinical pharmacist, condition-specific education and enhanced discharge planning by a care coordinator and phone follow‐up) could delay the time interval between first admission and readmission [19]. Using a similar tool, Cawthon et al. showed that over two-thirds of patients, especially those with illiteracy, found communication with a pharmacist to be very helpful [20].

In addition to its association with a higher risk of frequent readmission, initial longer LOS (top tertile) was also associated with increased risk of short-term, medium-term and long-term mortality in all ages. We also observed that patients who initially spent the longest time in hospital had the shortest time between discharge to death and shortest LOS during readmission. Longer LOS represents patients with complex medical and importantly social needs, irrespective of age. Studies have shown detrimental effects of prolonged bedrest in hospital. A loss of 5% of muscle strength each day of bedrest has been reported [21], while bedrest over 10 days is associated to the equivalent of 10 years of muscle ageing for individuals over 80 years [22]. Older patients are also at increased risk of hospital-acquired infections such as pneumonia and urinary tract infections [23], and infection rates have been estimated to occur at 0.6% per day in patients over 60 years [24]. Studies have also shown an association between longer stay in hospital and adverse drug reactions (ADR) [25, 26]. However, it is not certain whether prolonged LOS leads to an increased risk of ADR or *vice versa*. It is plausible that their relationship is bidirectional. The human cost of death imposes immense burdens on the patient’s family and society, particularly in young individuals, and results in profound social and economic consequences due to the loss of years of productivity and the effects on their household dependency [27].

### Implications for policy, practice and research

There remains uncertainty about whether early discharge (a short LOS) may be detrimental to patients. Early discharge has therefore been implicated, or even suspected, to be premature. Although we found no evidence to support this notion in this study, readmission rates vary widely between European countries and between US states [6]. This may be influenced by differences in patient characteristics including older age, index admissions, co-morbidities (especially those that are poorly managed), pharmacological agents, inadequately supported post-discharge care in the community [28], effective communication with primary care physicians [29] and socio-economic background [30]. The standard of clinical practice may differ between centres, particularly in different countries. At our hospital, the decision to discharge is usually taken by a senior clinician (consultant). Timely and safe discharge depends on the skill and experience of the clinical staff involved in the patient’s care, frequent communication with the patient and family together with close liaison with community agencies.

### Strengths and limitations

The strengths of this study lie in its large number of consecutive adult patients with a wide age range (18–107 years). This enabled us to estimate the risk of mortality by different categories of frequency and reason for readmission that has not been explored in previous studies, and with appropriate adjustments for age, sex, patients’ severity of underlying health status and index admissions. The population catchment area for this study is relatively affluent compared with the rest of the UK but their subject characteristics are similar to those of the overall UK population [8, 31]. There are certain limitations, including potential loss of patients who might have subsequently been admitted to another hospital or moved away from the area, so that rates of readmission or mortality could be underestimated. Other factors that may introduce a bias include those who developed a terminal illness and needed palliative care instead of hospital readmission and those transferred to rehabilitation for conditions such as stroke and hip fracture. The present study chose LOS cut-offs at tertile levels to obtain equal sample sizes for LOS categories, and thus minimized problems with heteroscedasticity [32]. However, we have also explored other cut-off thresholds, such as 1 and 2 weeks, which identify respectively the 20% and 10% of patients staying longest in hospital, and other thresholds such as those that correspond to 50th and 75th centiles, all of which showed similar patterns of association between LOS and outcomes, i.e. shortest LOS was associated with lowest risk of readmissions and mortality. We additionally adjusted our data for underlying health status since some conditions may be associated with longer LOS, readmissions and mortality than others, as well as different categories of CCI including trichotomising at 0, 1 and >1. However, the associations of LOS and outcomes were not significantly altered. The period of early emergency readmission has been variably defined between 28 and 30 days [33]. In the present study, we used 28 days for our definition based on NHS guidance [34]. It should be borne in mind that bed pressure varies between countries and may be more critical in the UK than other European countries such as France and Germany [35]. Our data were obtained just prior to the Covid-19 pandemic. Since then, LOS and outcomes have changed drastically during the pandemic [36]. Continuation of this kind of study is necessary to assess future changes to ensure maintenance and improvement of care-quality standards.

Additional limitations may arise from other factors that were not accounted for in the present study. Therefore, we cannot definitively deduce that differences in outcomes between LOS groups as a timely discharge since a natural consequence of the difference in severity of the two groups may be the underlying factor. We would therefore acknowledge that with the available data, it is not feasible to assess the proper impact of the LOS on the outcome.

## Conclusion

Short LOS in hospital was associated with less frequent hospital readmissions, a delay in time interval from discharge to death and shorter LOS in hospital during readmission, and lower risk of post-discharge mortality in adults from young to older age groups, independent of age, sex and patients’ severity. The findings indicates that short LOS does not equate premature discharge in this particular hospital, which meet the aim of GIRFT.

## Supplementary Material

mzab160_Supp

## Data Availability

The data underlying this article will be shared on reasonable request to the corresponding author.
