# Supplementary material for: Evaluation of the association of length of stay in hospital and outcomes
Source: Int J Qual Health Care. 2021 Dec 17;34(2):mzab160. doi: 10.1093/intqhc/mzab160 (PMC9070811; doi:10.1093/intqhc/mzab160)

**Supplementary Table 1.** Characteristics of 14 878 men aged 18.0-104.1 years (mean = 63.9 ±SD = 19.3 years) and 17 392 women aged 18.0-106.7 years (mean = 64.1 ±SD = 21.6years).

|  | n | % |
| --- | --- | --- |
| **Age bands** |  |  |
| 18-49 years | 8403 | 26.0 |
| 50-59 years | 4304 | 13.3 |
| 60-69 years | 4739 | 14.7 |
| 70-79 years | 6068 | 18.8 |
| ≥80 years | 8756 | 27.1 |
| **Readmissions within 28 days of discharge** |  |  |
| None | 28548 | 88.5 |
| 1 readmission | 2666 | 8.3 |
| ≥2 readmissions | 1056 | 3.3 |
| **Mortality status** |  |  |
| Death within 30 days of discharge | 834 | 2.6 |
| Death within six months of discharge | 2192 | 6.8 |
| Death within a two-year period | 3305 | 10.2 |
| **Index admissions** | n | % |
| All cardiac disorders | 4416 | 13.7 |
| *Myocardial infarct^✝^* | 796 | 2.5 |
| *Atrial fibrillation^✝^* | 399 | 1.2 |
| *Congestive heart failure^✝^* | 405 | 1.3 |
| Neurological disorders | 722 | 2.2 |
| All pulmonary disorders | 3367 | 10.4 |
| *Asthma^✝^* | 207 | 0.6 |
| *Chronic obstructive pulmonary disease^✝^* | 470 | 1.5 |
| *Pneumonia^✝^* | 1692 | 5.2 |
| Gastrointestinal disorders | 4144 | 12.8 |
| Endocrine disorders | 1084 | 3.4 |
| Rheumatological disorders | 3169 | 9.8 |
| Haematological disorders | 676 | 2.1 |
| Psychiatric disorders | 279 | 0.9 |
| Dermatological disorders | 1083 | 3.4 |
| All genitourinary disorders | 3686 | 11.4 |
| *Urinary tract infection^✝^* | 1116 | 3.5 |
| Sepsis | 1170 | 3.6 |
| Viral infections | 145 | 0.4 |
| Ophthalmic disorders | 163 | 0.5 |
| Non-specific bodily pain | 4344 | 13.5 |
| Bone fractures | 2624 | 8.1 |
| *Hip fractures^✝^* | 639 | 2.0 |
| Medical device-related complications | 1015 | 3.1 |
| Others (not specified) | 183 | 0.6 |

^✝^Specific conditions within categories of disorders.

**Supplementary Table 2.** Distribution of index admissions in relation to length of stay in hospital.

|  | Length of stay in hospital | | | Group differences | |
| --- | --- | --- | --- | --- | --- |
| **Index admissions** | Bottom tertile | Middle tertile | Top tertile | χ^2^ | *P* |
| Myocardial infarct | 0.4 | 4.6 | 2.4 | 395.4 | <0.001 |
| Congestive heart failure | 0.4 | 0.9 | 2.5 | 212..1 | <0.001 |
| Atrial fibrillation | 1.7 | 1.4 | 0.6 | 53.2 | <0.001 |
| Neurological disorders | 37.3 | 24.8 | 38.0 | 24.3 | <0.001 |
| Asthma | 0.5 | 1.1 | 0.4 | 42.5 | <0.001 |
| Chronic obstructive pulmonary disease | 0.9 | 1.7 | 1.8 | 38.1 | <0.001 |
| Pneumonia | 1.9 | 4.7 | 9.1 | 568.3 | <0.001 |
| Gastrointestinal disorders | 12.6 | 14.2 | 11.8 | 29.3 | <0.001 |
| Endocrine disorders | 27.0 | 44.7 | 28.2 | 65.9 | <0.001 |
| Rheumatological disorders | 7.3 | 13.8 | 8.4 | 291.7 | <0.001 |
| Haematological disorders | 2.7 | 2.4 | 1.1 | 75.3 | <0.001 |
| Psychiatric disorders | 0.6 | 0.6 | 1.4 | 62.5 | <0.001 |
| Dermatological disorders | 5.7 | 1.8 | 2.6 | 290.6 | <0.001 |
| Genitourinary disorders | 12.1 | 13.0 | 9.1 | 87.4 | <0.001 |
| Sepsis | 1.7 | 3.3 | 5.9 | 282.8 | <0.001 |
| Viral infections | 0.6 | 0.5 | 0.3 | 12.8 | 0.002 |
| Ophthalmic disorders | 1.0 | 0.3 | 0.2 | 88.7 | <0.001 |
| Non-specific pain | 24.3 | 9.5 | 6.7 | 1648.9 | <0.001 |
| Bone fractures | 5.9 | 6.3 | 12.2 | 355.1 | <0.001 |
| Medical device-related complications | 3.9 | 2.8 | 2.8 | 29.2 | <0.001 |

**Supplementary Table 3.** Number of patients exposed to risk.

| **Time (months)** | **0** | **5** | **10** | **15** | **20** | **25** | **30** | **35** |
| --- | --- | --- | --- | --- | --- | --- | --- | --- |
| **Bottom tertile of LOS** | 10754 | 10121 | 8483 | 6235 | 4141 | 2058 | 497 | 0 |
| **Middle tertile of LOS** | 10757 | 10021 | 8372 | 6097 | 3988 | 1987 | 484 | 0 |
| **Top tertile of LOS** | 10759 | 8903 | 7095 | 5039 | 3138 | 1438 | 320 | 0 |

**Supplementary Figure 1.** ROC curves to estimate the ability of hospital LOS to predict: a single readmission (**a**) and ≥2 readmissions within 28 days of discharge from hospital (**b**), as well as all-cause mortality (**c**).

(a)


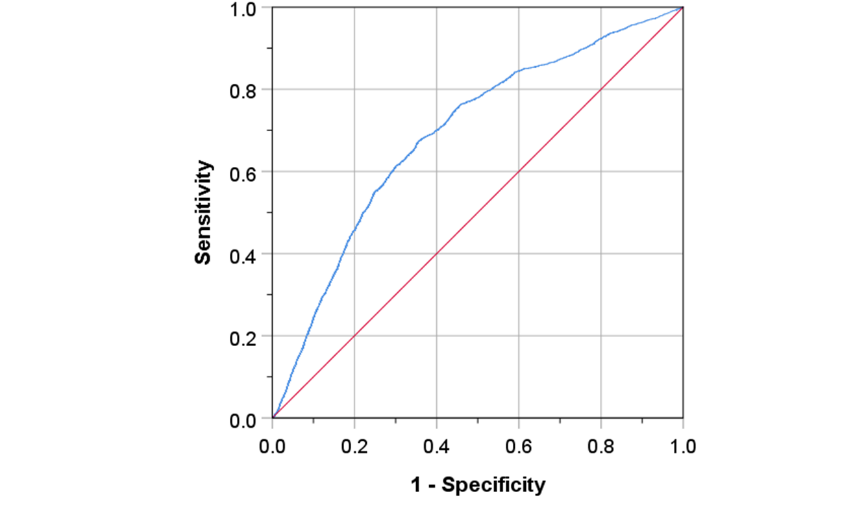


(b)


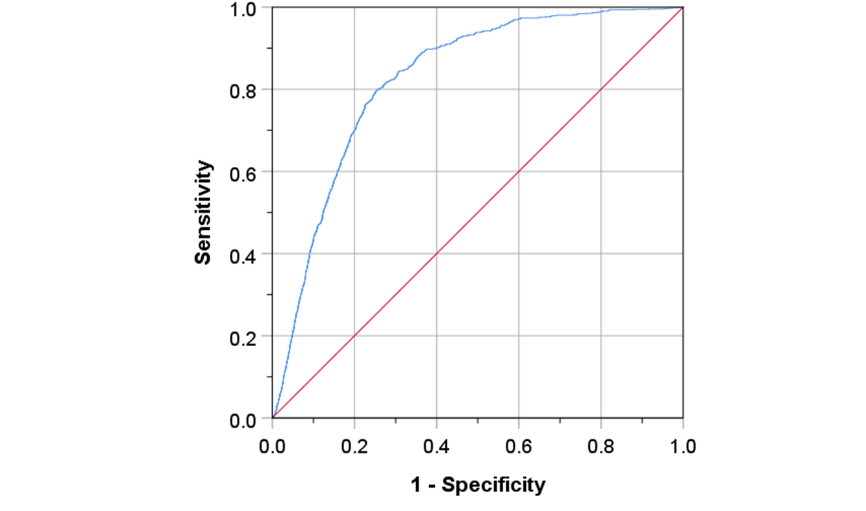


(c)


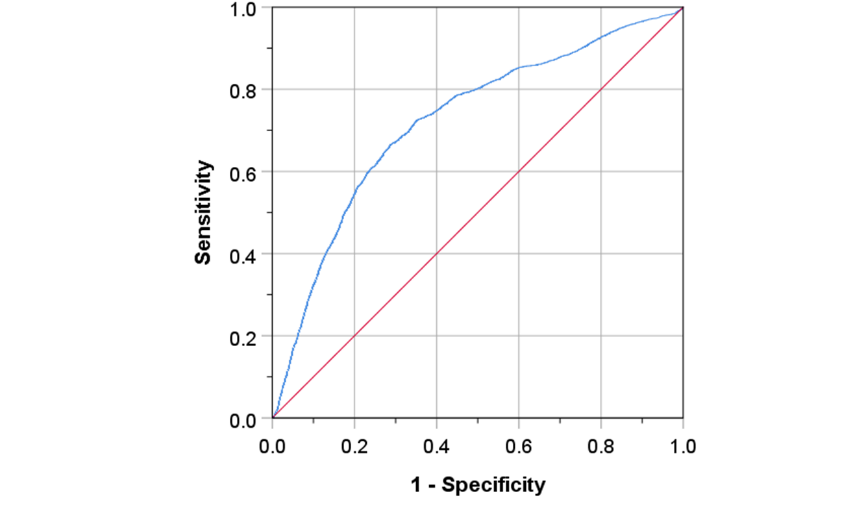

Supplement: mzab160_Supp [file mzab160_supp.zip › mzab160_Supp.docx]
